# Effects of emotion coaching group programme for mothers of preschool children with smart device overdependence: a mixed methods study

**DOI:** 10.1186/s12912-023-01554-9

**Published:** 2023-10-13

**Authors:** Gumhee Lee, Sungjae Kim

**Affiliations:** 1 College of Nursing, Seoul National University, Seoul, Republic of Korea; 2 College of Nursing, The Research Institute of Nursing Science, Seoul National University, 103 Daehak-ro, Jongno-gu, Seoul, 03080 Republic of Korea

**Keywords:** Smartphone, Behaviour, addictive, Child, preschool, Infant, Mothers, Emotional intelligence, Parenting, Object attachment

## Abstract

**Background:**

Smart device overdependence adversely affects the overall development of preschool children. This study aimed to provide an emotion coaching group programme to mothers raising preschool children who are overly dependent on smart devices and to confirm the programme’s effectiveness.

**Methods:**

This concurrent embedded mixed methods study used a non-equivalent control group pre-post design to verify the quantitative effect of the programme, and a descriptive research design to verify the qualitative effect. The study collected data from June 2019 to March 2021 from 51 (25 experimental, 26 control) mothers raising children aged 3–6 years in South Korea. The quantitative study examined the effects of an eight-session programme, conducted once a week for two hours, on mothers’ emotional intelligence, parenting behaviour, and their children’s attachment stability and smart device overdependence using a non-equivalent control group pre-post design. The analysis used a generalized estimate equation. For qualitative research, focus group interviews were used as a descriptive research design method, and content analysis was used for analysis.

**Results:**

Quantitatively, the results showed significant differences between the experimental and control groups in terms of changes in maternal emotional intelligence (χ^2^ = 14.99, p = .001), maternal positive parenting behaviour (χ^2^ = 9.32, p = .009), children’s unstable attachment to their mothers (χ^2^ = 9.49, p = .009), and children’s overdependence on smart devices (χ^2^ = 14.48, p = .001). Qualitatively, the study derived five themes for the experiences of mothers who participated in the programme: “hope for desirable parenting without emotional difficulties,” “feelings touched by reflection,” “warm but authoritative parenting,” “children who became responsible by gaining psychological stability,” and “changed view of my home.”

**Conclusions:**

The emotion coaching group programme was effective in improving the emotional intelligence and parenting behaviour of mothers raising preschool children. The programme also improved preschool children’s attachment stability to their mothers and overdependence on smart devices. The programme can be useful in community practice, and the present study can be used as evidence for education and research related to device overdependence.

## Background

### Rationale for the study

A smartphone is a type of portable computer and refers to a small device that can use wireless phone communication and application software (apps); a smart pad is typically considered a smartphone [1]. The term “smart device” includes non-portable smart TVs in addition to smartphones [1]. With the development of information and communication technology, most households use smart devices, including preschool children between the ages of 3 and 6 years [2, 3]. With the generalization of smart device use among preschool children, the level of smartphone overdependence has also increased; the risk of smartphone overdependence rate among preschool children in South Korea (hereinafter, Korea) reached 24.7% in 2022 [1].

The widespread use of smart devices even by preschool children has advantages in terms of education, entertainment, and parenting assistance [1, 4]. However, excessive use of smart devices causes sleep disorders in preschool children [5] and deformation of the musculoskeletal system [6]. It also adversely affects emotional regulation [7] and causes increased impulsivity and decreased concentration in daily life [8]. Moreover, the one-way communication method of smart devices has a negative effect on the formation of interpersonal relationships [9]. Excessive use of smart devices by children has been reported to mediate gambling addiction in adolescence in the long term [10].

Given that mothers are often the main caregivers of preschool children, their characteristics are more important than fathers’ as factors affecting smart device overdependence [11]. In particular, when the mother has low emotional intelligence, she may have difficulty providing positive parenting behaviour that is based on psychological stability and emotional sensitivity [12]. Thus, inconsistent negative parenting behaviours are provided, which hinder the formation of attachment stability in children [13]. Those who do not form attachment stability may show attachment reactions to highly accessible smart devices owing to chronic frustration of needs [12]. Poor supervision of the use of smart devices also increases overdependence [14]. Therefore, to reduce overdependence on smart devices in preschool children, mothers need to consider changes to their parenting behaviour.

Emotion coaching is a communication system that helps identify emotions and solve problems by sensitively recognizing and communicating with children’s emotions, and is based on understanding and regulating emotions [13]. Therefore, by improving mothers’ emotional sensitivity and psychological stability, emotion coaching can contribute to the enhancement of attachment stability and reduction of smart device overdependence in preschool children by inducing positive changes in emotional intelligence and parenting behaviour [15, 16].

Although a quarter of preschool children are already at risk for smart device overdependence [1], few studies have provided interventions to mothers of preschool children who are overly dependent on smart devices [17–19]. Therefore, we designed an emotion coaching group programme for mothers of preschool children who were overly dependent on smart devices and then assessed the programme’s effects on mothers’ emotional intelligence, parenting behaviour, preschool children’s attachment, and smart device overdependence. We used a mixed study design, whereby both quantitative and qualitative data are processed to generate meta-inferences [20], to closely understand the effects of the intervention.

### Objectives

We aimed to verify the effects of an emotion coaching group programme applied to mothers raising preschool children with smart device overdependence. Our specific objectives were as follows: to confirm the effects of the emotion coaching group programme on mothers’ emotional intelligence, parenting behaviour, preschool children’s attachment stability, and smart device overdependence; and to explore qualitatively the experiences of mothers who participated in the emotion coaching group programme.

### Conceptual framework and hypothetical model

Lee et al. [12] verified the relationship between mothers’ emotional intelligence, negative parenting behaviour, preschool children’s attachment instability, and smart device overdependence through a structural equation model study. The final model showed the following fit indices: χ^2^ = 22.023, degrees of freedom = 8, p = .005, χ^2^/df = 2.753, comparative fit index = 0.976 (≥ 0.9), normed fit index = 0.963 (≥ 0.9), standardized root mean squared residual = 0.0366 (≤ 0.1), root mean square error of approximation = 0.079 (0.040–0.12). In the model, four paths had significant standardized path coefficients. Mothers’ emotional intelligence had a direct negative relationship with mothers’ negative parenting behaviour (β = -0.44, p < .001). Mothers’ emotional intelligence had an indirect negative relationship with preschool children’s attachment instability (β = -0.25, p < .001) and smart device overdependence (β = -0.24, p < .001). Mothers’ negative parenting behaviour had a direct positive relationship with preschool children’s attachment instability (β = 0.56, p < .001) and both direct and indirect positive relationships with preschool children’s smart device overdependence (β = 0.55, p < .001).

**Fig. 1:**
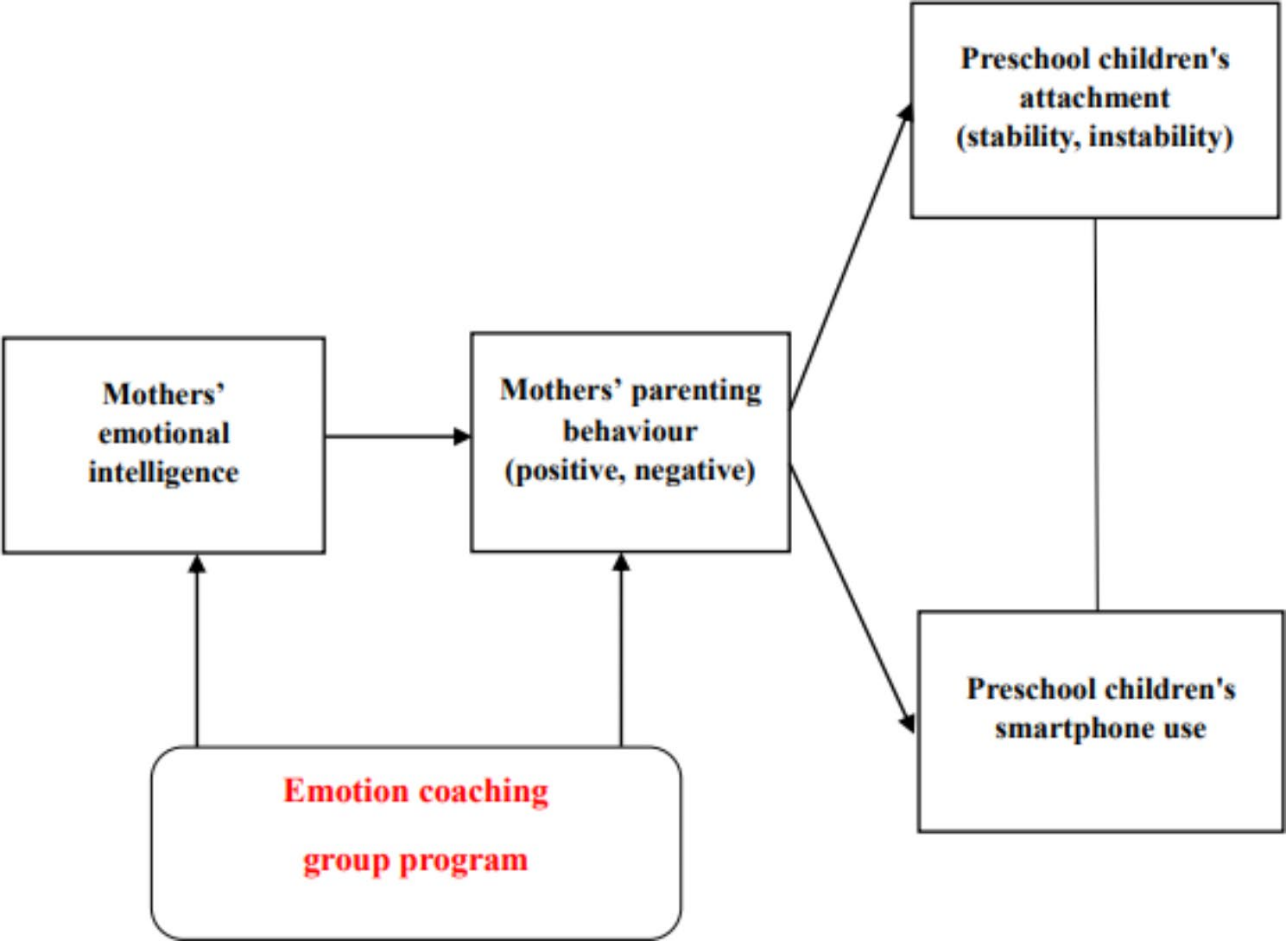
Conceptual framework of this study

The principle of emotion coaching is to sensitively recognize the child’s emotions and respond empathetically by enhancing the emotional capacity of the mother [13]. In addition, the principle of emotion coaching, which is limited to unacceptable behaviours, can improve mothers’ emotional intelligence [15, 16] and parenting behaviour [13]. According to previous studies, if the mother’s emotional intelligence and parenting behaviour are improved through the emotion coaching group programme, preschool children’s attachment and overdependence on smart devices can be expected to improve as a result [12, 13, 15, 16]. Therefore, in this study, the intervention was applied only to mothers, and the effect on children was thereafter observed. The conceptual framework of our study, based on previous studies, is shown in Fig. 1.

## Methods

### Study design

To evaluate the effect of the emotion coaching group programme, we used a concurrent embedded strategy of mixed-method research [21]. This method focuses on one data type and complements it with other data types. We confirmed the effect of the programme mainly through quantitative research. Effects that were difficult to grasp by quantification through measurement tools were then confirmed using qualitative research [21].

### Participants

The specific selection criteria were as follows: (1) mothers raising preschool children (3–6 years old) who scored 24 points or higher on the Smartphone Overdependence Scale (SOS) for preschool children [22] (measured including smart TVs in this study), and (2) those who self-judged that they could understand the survey and education provided by the emotion coaching group programme. The exclusion criteria were as follows: (1) those who started taking psychiatric medications within the past four weeks or who had a change in medication dosage, and (2) those who participated in other psychological interventions or programmes during the study period. Even if one of the selection criteria was not satisfied, this programme was conducted by reflecting the participants’ concerns on the occurrence of stigma and the desire of participants and early childhood education institutions with the desire to participate in the emotion coaching group programme in the community. Among the participants in the programme, the subjects of analysis were kept confidential; only the researchers and participants themselves could know. The sample size was calculated using G*Power 3.1 [23], setting the following parameters: effect size, 0.20; power, 0.80; significance level, 0.05; correlation coefficient, 0.5; RM ANOVA calculation results, 21 per group. We derived a total of 42; when the dropout rate was 30%, we set the number of participants per group at 30 (total of 60 people).

### Setting and intervention

The emotion coaching group programme applied in our study was based on participants recognizing and regulating their emotions. In addition, it aimed to help participants understand the five stages of emotion coaching and improve their application capabilities (Table 1). The programme was reconstructed based on the HD Happiness Institute’s five-step emotion coaching training course and previous studies that applied emotion coaching interventions to mothers [15, 16].

**Table 1:** Contents of the Emotion Coaching Group Programme

Session	Theme (Each theme is applied for 2 h every week)	Performance objective Participants will:	Contents	Method
1	What is emotion coaching?	• Increase their understanding of the programme • Increase their willingness to participate in the programme	• Pre-test • Introduction of group programme progress and facilitators • Importance of emotions • Definition and principles of emotion coaching • Set collective rules • Sharing motivation for participation and expectations for the programme	Small-group lecture and talk
2	How about my parenting style?	• Identify their parenting style • Understand the impact of meta-emotion on parenting • Practice the three-step self-soothing method	• Types of parenting styles and own parenting styles • Relation between meta-emotion and parenting style • Meta-emotion check • Three-step self-regulation (heart breathing) • Task: emotion diary to maintain composure, exercise	Small-group lecture and practice
3	Emotion coaching step 1_ Be aware of your child’s emotion	• Verbally express the relation between attachment loss and smart device overdependence • Express the confidence to apply step 1 of emotion coaching	• Importance of attachment • Side effects of overdependence on smart devices • Play for attachment formation • Step 1 of emotion coaching • Meaning of “accept all the child’s feelings but modify the behaviour” • Various human emotions and their importance • Capturing emotions through facial expressions (practice)	Small-group lecture Group activity
4	Emotion coaching step 2_ Recognize your child’s expression of emotion as a perfect moment for intimacy and teaching	• Understand the developmental characteristics of preschool children • Appropriately apply the second stage of emotion coaching to the situation	• Developmental and behavioural characteristics of preschool children • Step 2 of emotion coaching • Sharing moments of emotions you want to avoid, capturing coaching moments • Skills required for emotion control (control of volume, tone, and speed of voice)	
5	Emotion coaching step 3_ Listen with empathy and validate your child’s feelings	• Practice by understanding the attitude for step 3 of emotion coaching • Participants understand and apply the delivery method	• Step 3 of emotion coaching • Importance of empathy and the skill of active listening in emotion coaching • I-massage (practice)	Small-group lecture Group activity
6	Emotion coaching step 4_ Help your child learn to label their emotions with words Emotion coaching step 5_ Set limits when you are helping your child solve problems or deal with upsetting situations appropriately	• Understand and apply steps 4 and 5 of emotion coaching	• Effect of step 4 of emotion coaching • Helping children express their emotions • Effects of five steps of emotion coaching • How to apply the five steps of emotion coaching using role play • Unsuitable situations during emotion coaching application	Small-group lecture Group activity
7	Intensive emotion coaching training	• Identify and improve the factors that cause difficulty in applying the five steps of emotion coaching	• Desirable emotion coaching practice strategy • Comprehensive application of the five steps of emotion coaching	Small-group lecture Group activity
8	I’m a good mom	• Discover yourself growing through emotion coaching and design a positive future	• Preparation for change (my life curve viewed with emotion, what I felt through the programme, what changed, words of praise to me, hopes and commitments for the future) • Resilience • Sending thanks to one another • Termination of group programme • Post-test	Group activity

The title of the programme was set as “Can my precious child become a child with many friends,” emphasizing the expectation that reducing overdependence will contribute to improving peer relationships [24], without using the term “overdependence on smart devices” to prevent stigma. The details of the specific programme are given in Table 1. The type, duration, period, and group size of the programme provided to the experimental group were determined by comprehensively considering the community environment based on previous emotion coaching group programmes [25]. Finally, the semi-structured intervention was performed as a group of eight sessions for 120 min once a week, and the number of people per group was set from a minimum of 4 to a maximum of 12 for smooth group operation. There was no intervention in the control group. As for the group allocation, those who could participate in the intervention at the time of recruitment participated in the experimental group, and those who wished to participate in the waiting list or control group were assigned to the control group. The experimental group and the control group were divided based on the preferences of the organization and participants. However, the possibility of contact between subjects was minimized by recruiting the experimental and control groups from different institutions or individually online. The intervention occurred in a space where independence was guaranteed, and in the case of an interactive real-time video programme, participants participated from their homes. The intervention was provided by one researcher, who has the following credentials: Psychiatric-Mental Health Advanced Practice Nurse, Psychiatric-Mental Health Nurse Practitioner, Certified Addictions Registered Nurse, and Emotion Coach.

### Questionnaire

For general characteristics data, we analysed the participants’ age, level of education, religion, household income, and smart device usage time (both mothers and children). For preschool children’s smartphone overdependence, we used the nine-item SOS for children aged 3 to 9 years developed by the Korea Internet & Security Agency [22]. The items were rated on a four-point Likert scale ranging from *not at all* (1 point) to *very much so* (4 points). We observed the following scores and corresponding risk groups: 23 points or less, general user group; 24–27 points, potential risk group; and 28 points or more, high-risk group. Three items corresponding to control failure were reverse scored. In our study, Cronbach’s α was 0.85 overall and ranged from 0.68 to 0.88.

We also used the Adult Emotional Quotient Test (AEQT) used by Moon [26] based on the emotional intelligence model of Salovey and Mayer [27]. This tool has 45 items: 37 items are scored on a three-point Likert scale measured as *not so* (1 point), *sometimes likely* (2 points), and *always likely* (3 points); for 8 items, the respondent selects the item that they think is the most similar among the two items. The total score ranges from 45 to 127, with a higher score indicating higher emotional intelligence. In our study, Cronbach’s α was 0.86.

For parenting behaviour, we used the Maternal Parenting Behaviours Questionnaire (MPBQ) modified and adopted by Choi [28]. This scale consists of 23 items, among which 13 are on positive parenting behaviour and 10 on negative parenting behaviour [28]. Respondents are asked to respond on a five-point Likert scale ranging from *not at all* (1 point) to *very much so* (5 points), with higher scores indicating that mothers reported more of the behaviour when raising their children. In our study, Cronbach’s α was 0.79 for positive parenting behaviour and 0.62 for negative parenting behaviour.

We also used the Mother–Child Attachment Stability (MCAS) modified and adapted by Lee [29]. This scale consists of 24 items: 12 items on attachment stability and 12 on attachment instability. The MCAS instrument consists of a four-point Likert scale ranging from *not at all* (1 point) to *very much so* (4 points), and evaluates preschool children’s attachment to their mothers based on the mother’s observations of her child’s behaviour [29]. In our study, Cronbach’s α was 0.73 for attachment stability and 0.73 for attachment instability.

### Semi‑structured interview

Among the participants in the experimental group, those who understood the purpose of the qualitative research and voluntarily agreed to participate were invited to interview. We obtained written or online consent from the interview participants. The focus group interviews used a semi-structured questionnaire but minimized the number of questions to minimize the researcher’s effect on the results. The main questions were as follows: ‘What motivated you to participate in the emotion coaching group programme?’ and ‘What are the emotions, thoughts, and behaviours that have changed in relation to the use of smart devices?’

### Data collection

**Fig. 2:**
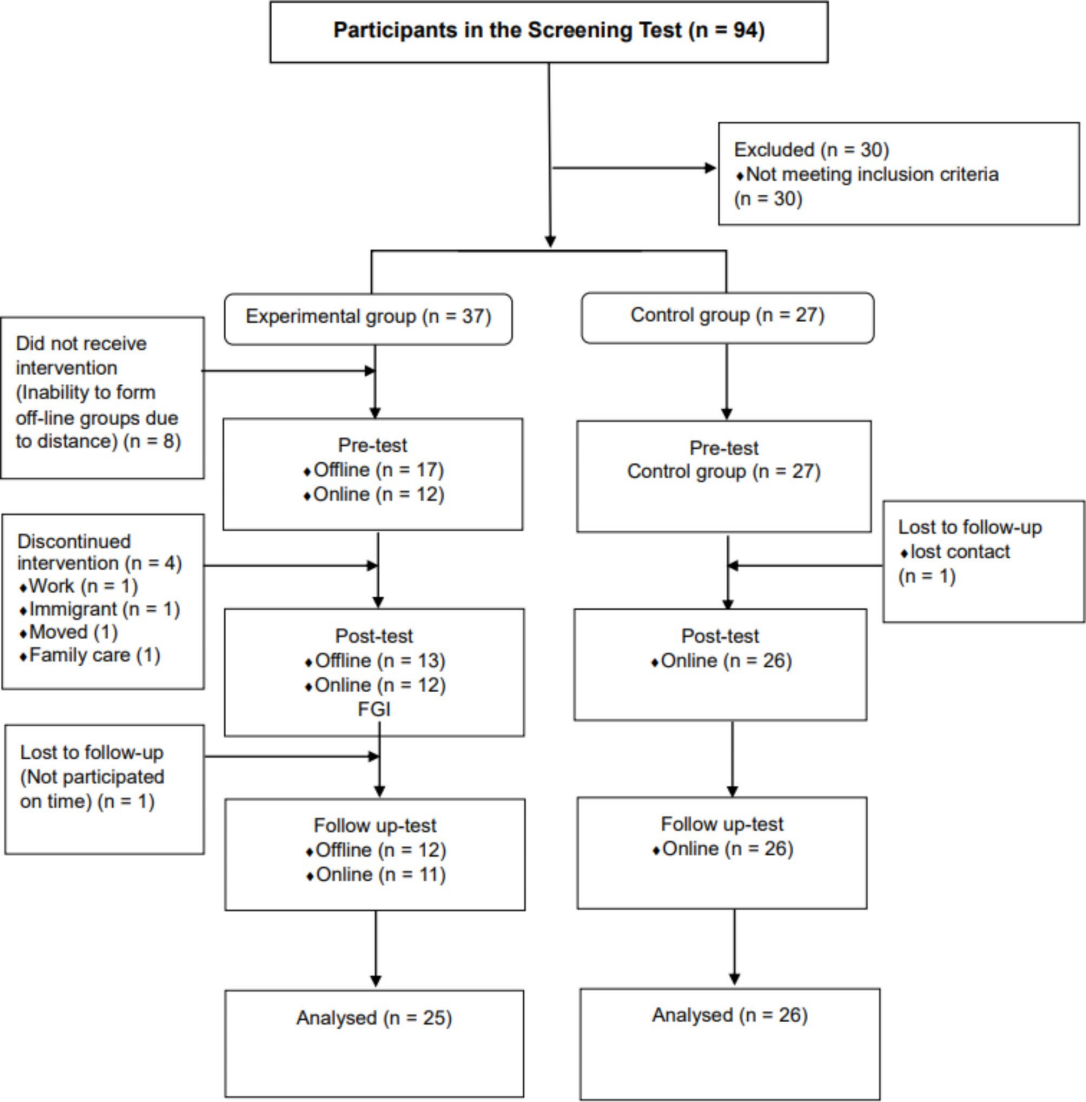
Flow diagram of participant recruitment and intervention process

From July 2019 to December 2020, we introduced the purpose of this study to managers of 60 kindergartens and day-care centres in Seoul and Gyeonggi-do, Korea, and four online ‘mom cafes’, or forums operated to share parenting information. Among them, four kindergartens and day-care centres, one church, and three online mom cafes (Bunta, Mamma Mia, Ilsan Ajimae), which allowed the promotion of this study, promoted this study and programme and recruited those who wished to participate voluntarily. The institution’s manager was not involved in the programme’s participation other than publicity. The intervention, which was conducted face-to-face from September 2019 to January 2020, was converted to an interactive real-time video programme from November to December 2020 owing to the COVID-19 pandemic. Four teams participated in the face-to-face programme, and four additional teams participated in the interactive real-time video programme, for a total of eight teams. In this study, to maintain the quality of the intervention, a semi-structured intervention comprising the same content was delivered by one researcher. The experimental group consisted of 25 people (13 offline participants and 12 interactive real-time video programme participants), and the control group had 26 participants (Fig. 2). For the experimental group, we conducted a pre-test before the programme, a post-test immediately after the end of the eighth session, and a follow-up test four weeks after the end of the programme. The control group was also tested at the same time and interval as the experimental group. Data were collected offline for the pre and post-test. We used an online questionnaire (Google Forms) for the follow-up test. Both the interactive real-time video programme and control group data were collected through Google Forms.

The recommended number of participants for a focus group interview is six to eight people [30]. However, it was difficult to organize a focus group with many people because the intervention end time of each team in the experimental group was different. Therefore, to collect the maximum amount of data, interviews were conducted if more than two interviewees per group could be recruited. In the end, 12 of the 25 participants in the experimental group voluntarily participated in the qualitative study. The qualitative interview was conducted by the researcher who had participated in several qualitative studies. Individual interviews for qualitative data collection were conducted immediately after the intervention, and each interview took about one hour.

### Ethical considerations

Our study was conducted in compliance with the Declaration of Helsinki and with the approval of the institutional review board of Seoul National University (IRB No. 1906/003–011). After explaining the purpose and procedure of this study, we selected only those who wished to participate voluntarily as study subjects. Instructions and informed consent were written in a language that the subjects could fully understand, and the purpose and procedure of the study were explained to the subjects prior to data collection. We explained to the research subjects that privacy and confidentiality were ensured through questionnaire coding, that participation in the study was not compulsory, and that they could give up participation at any time. We obtained written or online consent using a Google Form.

### Data analysis

#### Quantitative analysis

The collected data were analysed using IBM SPSS Statistics for Windows, Version 23.0. Chi-squared test and *t*-test were used to test the pre-homogeneity of the general characteristics and dependent variables of the experimental group and control group. Fisher’s exact test and Mann–Whitney U test were used if normality was not observed. The smart device overdependence data showed left-skewed normality because only mothers raising children with a score of 24 or higher were recruited. In addition, owing to missing persons in the follow-up test, the difference in change between groups over time was tested using the generalized estimation equation (GEE). The significance level of all statistics was set at p < .05 in a two-tailed test.

### Qualitative analysis

Qualitative data were analysed using the content analysis method of Elo and Kyngäs [31]. Given the scarcity of qualitative research on smart device overdependence, we first analysed it using an inductive analysis method by referring to the proposal of Elo and Kyngäs [31]. Second, based on our conceptual framework, we reconstructed the analysis results with a deductive analysis method to confirm the contents corresponding to the changes in the dependent variable. Our content analysis method included preparation, organization, and reporting stages [31]. In the preparation stage, the text was read repeatedly from beginning to end, and similar contents were collected and classified into subtopics. In the organizing stage, based on the literature review, we developed a matrix that categorized mothers’ emotional intelligence, parenting behaviour, preschool children’s attachment, and smart device overdependence into sub-domains. The main categories and sub-domains were identified through the categorical matrix. Next, the text was read repeatedly, and meaningful words and sentences were selected and coded appropriately for the matrix. In the final report stage, we analysed the effects of the emotion coaching group programme on mothers and children in each matrix, and then reconstructed and described meanings as themes.

To secure the validity of our qualitative research, we checked the reliability, suitability, auditability, and confirmability of the data [32]. To increase the reliability of the data, we collected data from as many participants as possible, recorded and memorized the contents of the focus group interview, and summarized the participants’ story immediately after the interview and confirmed the accuracy of the statements. Various experiences were expressed through open-ended questions, and participants were directly quoted from the transcripts of the interviews. To establish suitability, we analysed only the data of research subjects who belonged to the selection criteria. The data collection method and analysis procedure were described in detail to establish auditability. To ensure confirmability, we confirmed the obtained results with participants to ensure that the statements were congruent. We received criticism and advice on the entire data analysis process from a nursing professor with extensive experience in qualitative research.

## Results

### General characteristics

The average age of the 51 participating mothers was 36.90 ± 4.00 years, most of whom had children aged five years. The amount of time spent using smart devices was an average of 2.5 h per day for both mothers and children. We found no significant difference in the general characteristics between the experimental and control groups, confirming the prior homogeneity of the two groups (Table 2).

**Table 2:** Homogeneity Test of General Characteristics of the Mothers between the Two Groups at Baseline(N = 51)

Characteristics	Categories	Exp. (n = 25)	Con. (n = 26)	t/z/ χ^2^	*P*
		n (%) or M (SD)	N (%) or M (SD)		
Mother’s age		36.28 (4.84)	37.50 (2.97)	-1.08	0.286
Education	< High school Vocational college College graduate Over graduate school	2 (8.0) 9 (36.0) 11 (44.0) 3 (12.0)	2 (7.7) 5 (19.2) 12 (46.2) 7 (26.9)	2.81^*^	0.434
Occupation	Homemaker Full-time job Part-time job Unemployed	11 (44.0) 6 (24.0) 5 (20.0) 3 (12.0)	14 (53.9) 6 (23.1) 3 (11.5) 3 (11.5)	0.97^*^	0.888
Religion	Yes No	10 (40.0) 15 (60.0)	13 (50.0) 13 (50.0)	0.52	0.473
Smart device usage time per day, hours (SD)		2.3 (2.2)	2.8 (2.1)	-1.11^†^	0.267
Monthly income (10,000 KRW)	< 200 201–400 401<	0 10 (40.0) 15 (60.0)	0 11 (42.3) 15 (57.7)	0.16	0.870
Child’s sex	Girls Boys	13 (52.0) 12 (48.0)	11 (42.3) 15 (57.7)	0.48	0.488
Age (years)	3 4 5 6	4 (16.0) 7 (28.0) 11 (44.0) 3 (12.0)	2 (7.7) 5 (19.2) 12 (46.2) 7 (26.9)	3.85^*^	0.277
Birth order	First Second Third<	20 (80.0) 3 (12.0) 2 (8.0)	18 (69.2) 7 (26.9) 1 (3.9)	2.03^*^	0.456
Purpose of use (multiple responses, n = 104)	Education Recreation Babysitting Begging Including all	7 (15.6) 23 (51.1) 7 (15.6) 8 (17.7) 45 (100)	12 (20.3) 26 (44.1) 13 (22.0) 8 (13.6) 59 (100)		
Smart device usage time per day, hours (before participation)		2.3 (1.7)	2.7 (1.5)	-1.36^†^	0.175

^*^Fisher’s exact test; ^†^Mann–Whitney test; Con. = Control group; Exp. = Experimental group; KRW = Korean Won; SD = Standard deviation

### Comparison of the groups’ mean scores before and after intervention

We found no statistically significant difference (p > .05) in the outcome variables of mothers’ emotional intelligence, parenting behaviour, preschool children’s attachment, and smart device overdependence scores, confirming that the two groups were homogeneous (Table 3).

**Table 3:** Homogeneity Test of the Outcome Variables

Variables	Exp. (n = 25)	Con. (n = 26)	t/z	*p*
	Mean ± SD	Mean ± SD		
Preschool children’s smart device overdependence	26.56 ± 2.96	27.54 ± 2.83	-1.40^†^	0.160
Mothers’ emotional intelligence	96.04 ± 10.03	96.15 ± 9.16	-0.04	0.966
Mothers’ positive parenting behaviour	44.36 ± 5.72	47.08 ± 6.05	-1.65	0.106
Mothers’ negative parenting behaviour	27.16 ± 4.09	28.04 ± 4.63	-0.72	0.477
Preschool children’s stable attachment	3.16 ± 0.42	3.11 ± 0.42	-0.65^†^	0.514
Preschool children’s unstable attachment	2.09 ± 0.41	2.04 ± 0.51	0.42	0.674

Con. = Control group; Exp. = Experimental group; SD = Standard deviation. ^†^Mann–Whitney test

### Programme effectiveness

The results of the GEE performed to confirm the difference in study variables over time between the control and experimental groups were as follows. First, we found a significant group and time interaction effect of preschool children’s smart device overdependence (χ^2^ = 14.48, p = .001) (Table 4). Through the theme of “changed view of my home” derived through qualitative analysis, we confirmed that the level of actual use of smart devices by preschool children decreased as psychological stability and satisfaction increased through emotion coaching-type communication and play by mothers. Play based on mothers’ psychological stability was a factor contributing to the satisfaction of preschool children’s needs (Table 5).

**Table 4:** Comparison of the Preschool Children’s Smart Device Overdependence between the Two Groups

Variables	Group	Pre	Post	Follow Up	Source	χ^2^	*P*	Difference (Post-Pre)			Difference (Post 4weeks-Pre)		
		M ± SE	M ± SE	M ± SE				M ± SE	χ^2^	*p*	M ± SE	χ^2^	*p*
Smart device overdependence	Exp. (n = 25)	26.56 ± 0.58	18.36 ± 0.76	18.90 ± 0.85	G T GxT	17.94 87.82 14.48	< 0.001 < 0.001 0.001	-8.20 ± 0.71	12.63	< 0.001	-7.66 ± 0.73	11.25	0.001
	Con. (n = 26)	27.54 ± 0.55	24.23 ± 1.08	24.15 ± 1.09				-3.31 ± 1.18			-3.39 ± 1.04		
Mothers’ emotional intelligence	Exp. (n = 25)	96.04 ± 1.97	103.56 ± 2.01	104.08 ± 2.20	G T GxT	3.25 25.62 14.99	0.072 < 0.001 0.001	7.52 ± 1.22	13.85	< 0.001	8.04 ± 1.37	12.54	< 0.001
	Con. (n = 26)	96.15 ± 1.76	96.46 ± 1.69	97.57 ± 1.72				0.31 ± 1.51			1.42 ± 1.27		
Mothers’ positive parenting behaviour	Exp. (n = 25)	44.36 ± 1.12	49.36 ± 1.18	49.39 ± 1.36	G T GxT	0.51 34.27 9.32	0.474 < 0.001 0.009	5.00 ± 0.97	7.17	0.007	5.03 ± 1.05	0.79	0.376
	Con. (n = 26)	47.08 ± 1.16	48.65 ± 1.31	50.74 ± 1.23				1.57 ± 0.83			3.66 ± 1.13		
Mothers’ negative parenting behaviour	Exp. (n = 25)	27.16 ± 0.80	23.64 ± 0.91	24.20 ± 0.90	G T GxT	4.74 15.70 4.79	0.029 < 0.001 0.091	-3.52 ± 0.93	4.02	0.045	-2.96 ± 0.86	3.64	0.056
	Con. (n = 26)	28.04 ± 0.89	27.04 ± 0.97	27.17 ± 1.00				-1.00 ± 0.84			-0.87 ± 0.69		
Preschool children’s attachment stability	Exp. (n = 25)	3.16 ± 0.08	3.36 ± 0.08	3.31 ± 0.06	G T GxT	0.52 20.05 2.46	0.470 < 0.001 0.292	0.20 ± 0.05	1.29	0.257	0.15 ± 0.08	0.01	0.937
	Con. (n = 26)	3.11 ± 0.08	3.23 ± 0.08	3.27 ± 0.08				0.12 ± 0.06			0.16 ± 0.04		
Preschool children’s attachment instability	Exp. (n = 25)	2.09 ± 0.08	1.86 ± 0.07	1.80 ± 0.08	G T GxT	2.55 4.41 9.49	0.110 0.110 0.009	-0.23 ± 0.07	4.93	0.026	-0.29 ± 0.09	9.04	0.003
	Con. (n = 26)	2.03 ± 0.10	2.04 ± 0.08	2.16 ± 0.11				0.01 ± 0.08			0.13 ± 0.11		

Con. = Control group; Exp. = Experimental group; G = Group; M = Mean; SE = Standard error; T = Time.

**Table 5:** Theme on the Experience of Emotion Coaching Group Programme

Theme	Sub-theme	Contents
Hope for desirable parenting without emotional difficulties	Emotional challenges experienced from the attempt to control smartphone device use	Concerns about increased smartphone use Parenting behaviour characterized by anger and threats Repeated cycle of anger, guilt, and regret
	Wish to not pass down emotional distress	Frequently experienced anxiety and anger Parenting style resembling my own parents’ Emotional distress that should not be passed down
Feelings touched by reflection	Changes in the awareness of my and my child’s feelings	Newly discovered value of feelings Ignoring the child’s feelings because of their age Feelings that had been suppressed for considering them weak and inappropriate
	Being able to empathize with the child’s feelings through effort	Letting go of the obsession over parenting skills Intentional effort to empathize with the child Increased empathy while listening to the child Understanding the child’s feelings through role play
	Being able to perceive the value of self by paying attention to feelings	Guilt arising from practice that deviates from what has been learned My values that had only been perceived by performing my roles Feeling sorry for myself for neglecting my feelings Perceiving unconditional self-worth Acknowledging that I am already a good mother Beginning to take care of self through compassion “Feeling journal” helped with rational processing of feelings
	Needs expressed based on recognition of feelings	Feelings accepted as is without suppressing or minimizing them Problems being solved based on feelings
Warm but authoritative parenting	Warm parenting focused on emotion	Acknowledging the child as a unique human being with dignity Expressing positive feelings more actively Prioritizing the child’s care over house chores Applying emotion coaching to my care
	Mother’s authority established	Reflecting on the double standards for smartphone use Adjusting smartphone limits in consideration of the child’s opinions Strengthening emotion coaching competence using supervision
Children who became responsible by gaining psychological stability	Child’s stability and rich emotional expression	Less frequent crying and tantrums Child’s softer language Increased use of feeling words and expression of affection
	Child’s voluntary and responsible behaviours	Cleaning up voluntarily Child’s taking responsibility of their decisions Looking after sibling voluntarily
Changed view of my home	Playing with mom replaces smart device use	Playing with mom in place of using a smart device Playing with mom becomes more fun Increased creative play
	More peaceful home	Home free of yelling and crying

*Now that I listen to my child’s feelings, my child does not turn on the TV. Just doesn’t turn on the TV. My child watches TV because of boredom and having nothing to do. But I played some games with my child like Jenga when we were alone. That was just a little bit of time but I guess that kind of satisfied my child’s needs. (Participant 10)*

As psychological stability increased, mothers’ shouting and children’s crying decreased, and the house became calm and quiet.

*I kind of feel like I’ve become calm. Like, once I started to accept everything (feelings), I just became calm, and didn’t have anything to get mad about. Actually not that I didn’t have anything to get mad about but my threshold for being mad changed. My kids just do their stuff on their own. So, we decided that they’re going to just watch one more content, they would turn it off after that and put it back. And when they clean up, it’s not like they do it because they’re forced by mom… so they seem a little calm. The entire house seems calm. (Participant 4)*

Second, we found a significant group and time interaction effect of mothers’ emotional intelligence (χ^2^ = 14.99, p = .001) (Table 4). In the theme of “feelings touched by reflection,” the participants understood the importance of emotions that they had not known until then, and recognized that children, in particular, were neglectful of emotions when young (Table 5).

*I think I’m learning about the value of feelings. (Because I did not know the value of feelings,) I did not take care of my feelings and didn’t consider other people’s feelings as important either and so I hurt them. (Anonymous)*

Through participation in the programme, the participants did not focus on the children’s undesirable ‘behaviours’ but rather understood and satisfied the behavioural needs while identifying the ‘emotion’ behind it.

*I think I never asked my child why they wanted to keep watching (TV). “Why do you want to keep watching when you promised me not to?” They said, “I’m bored because you won’t play with me.” So, I asked once, “Oh, then how should I play with you?” “I want you to watch me when I play with the blocks.” So, I said, “Ok, then let’s do that,” and he would gladly do so. (Participant 9)*

Third, the effect of parenting behaviour on mothers was as follows. We found a significant group and time interaction effect on mothers’ negative parenting behaviour (χ^2^ = 4.79, p = .091) but not on mothers’ positive parenting behaviour (χ^2^ = 9.32, p = .009) (Table 4). The theme ‘warm but authoritative parenting’ derived through qualitative analysis showed the participants’ practice of compassionate parenting based on the ability to control and express their own and children’s emotions in a desirable manner. In addition, even in the case of neglectful participants who did not have any restrictions on the use of smart devices, as the mother gained authority through clear expression of opinion based on psychological stability, the child’s begging behaviour decreased, tending to ask the mother for permission instead (Table 5).

*Since she can see the time on the smartphone, I talk with my child to agree with the time limit, like “The limit is 30 minutes, so you can play until 5:50,” and I have my child keep watch on the time and turn it off after that time. I was surprised that my child actually observes the time limit. I thought it wouldn’t work. Sometimes, my child goes over by 2, 3 min while watching a video, and when that happens, they would tell me, “Mom, I’ll just finish this video. I’m sorry.” I was so amazed. (Participant 1)*

Fourth, we found no significant group and time interaction effect of preschool children’s attachment stability (χ^2^ = 2.46, p = .292). However, we noted a significant difference in the measurement period and group interaction for attachment insecurity in preschool children (χ^2^ = 9.49, p = .009) (Table 4). For the theme ‘children who became responsible by gaining psychological stability’ derived through qualitative analysis, we found that the behaviour of children changed as participants applied new parenting behaviours. In addition, children with increased sense of security appeared to take responsibility for their own behaviours (Table 5).

*I changed my daily language after participating in the programme, and after I changed my language, my child’s language softened as well. My child was given rules, and I consoled them in my arms, so their crying voice changed. My child laughs and seems to have become more stable. (Participant 6)*

*I’m so amazed. Before, I had to force my child and say, “Clean up!” But now, I tell my child, “Let’s clean up together later. Let’s clean up after I finish washing the dishes, ok?” And my child would start cleaning up while I’m doing the dishes. (Participant 4)*

## Discussion

Our study, by applying a mixed research method, confirmed that the emotion coaching group programme provided to mothers changed the mothers’ emotional intelligence and parenting behaviour, as well as the attachment of these mothers’ preschool children, which are factors related to smart device overdependence in preschool children.

### Effects on reducing smart device overdependence

Compared with the control group, the children of the experimental group participants showed a significant decrease in smart device overdependence scores over time. Making direct comparisons prove difficult because there are few intervention studies that employ tools with tested reliability and validity for identifying and recruiting preschool children who are already overdependent on smart devices, while focusing on reducing their subsequent overdependence on smart devices. However, this study shows similar results to those that confirmed significant positive changes in smart device usage. These studies employed cognitive behavioural therapy-based interventions targeting both preschool children and their mothers [17] or only preschool children [18], belonging to the general user group—a group encompassing individuals not overdependent on smart devices. Therefore, our results show that the emotion coaching group programme is effective in reducing children’s overdependence on smart devices, and it can be inferred that it is an effective intervention that can be used as an alternative to cognitive behavioural therapy.

Behaviour addiction, like substance addiction, tends not only to repeat the addictive behaviour but also to gradually increase in intensity [33]. This study is meaningful as it confirms its effectiveness beyond the secondary prevention among preschool children who have already developed overdependence. Through the ‘changed view of my home’, a theme derived through qualitative analysis, the effect of reducing overdependence on smart devices was maintained one month after the intervention ended. As the children got used to playing with their mothers, they gradually developed their play in a ‘creative way’. Thus, the participants acquired desirable coping strategies and techniques to reduce their preschool children’s overdependence on smart devices. Given that the use of smart devices by preschoolers is rapidly increasing [1], emotion coaching group programmes for mothers targeting changes in their children are beneficial due to their high accessibility and facilitation of more profound changes. The next sections discuss the impact of the emotion coaching group programme on each variable.

### Effects on mothers’ emotional intelligence improvement

We found significant differences in emotional intelligence over time between the experimental and control groups. These results are supported by previous findings that the emotion coaching group programme can contribute to the improvement of emotional intelligence in mothers of preschool or school-age children [15, 16].

In the theme ‘Hope for desirable parenting without emotional difficulties’ derived through qualitative analysis, most of the participants experienced negative emotions such as anxiety or anger in the attempt to control their children’s use of smart devices. As a result, the participants attempted to limit such usage by shouting and threatening, but this was not an effective approach. This suggests that participants’ negative perceptions of excessive use of smart devices caused negative emotions because, as reported by Liu et al., focusing on problems can increase negative emotions—such as anxiety—by anticipating negative outcomes [34]. In conclusion, this study confirmed that the participants’ negative perception of their children’ excessive use of smart devices caused negative emotions and led to negative parenting behaviours. This is in line with the concept that negative emotions can lead to negative parenting behaviour if the mother is not accustomed to effective communication and is unable to control her negative emotions [12]. However, mothers’ negative parenting behaviour has a direct and indirect relationship with preschool children’s impaired attachment and increased overdependence on smart devices through inadequate supervision [12].

The qualitative findings of this study suggest that it is difficult to reduce smart device overdependence in preschool children through haphazard efforts alone, and that interventions systematically designed by experts are needed. This study demonstrates that intervention providers’ assessment of mothers’ attitudes and emotions towards smart device overdependence is an important antecedent to developing interventions to reduce smart device overdependence. Additionally, the results show that the mother should improve her emotional intelligence, which is a variable that represents her emotional capacity, so that she can recognize and regulate her emotions.

In this study, we selected ‘emotions’ as an important element influencing smart device overdependence and focused on those experienced by mothers and their children in parenting situations, rather than the addictive ‘behaviours’ themselves. The results of the qualitative analysis showed that, by focusing on their own and their children’s emotions, participants were able to reduce their obsession with their children’s excessive use of smart devices and pay attention to their children’s needs. Furthermore, as the participants’ understanding of emotions increased, they were able to improve their self-regulation of emotions and continue to exhibit compassionate and authoritative positive parenting behaviours towards their preschool children. This is consistent with Koo and Kwon’s conclusion that focusing on emotions and increasing basic understanding of the needs underlying children’s behaviour can help regulate negative emotions [16]. In this way, the emotion coaching group program provided in this study significantly improves mothers’ emotional intelligence, which can be interpreted as contributing to changes in parenting behaviour and, consequently, improving preschool children’s overdependence on smart devices.

When examining the experience of the participants in detail using the results of qualitative analysis, we found that the participants first recognized the importance of their children’s emotions, which had been dismissed or suppressed, considering them insignificant because the children were young. Thus, the immediate negative reaction to children’s behavioural problems or emotional expression stopped. In addition, through conscious effort, the mothers learned to empathize with their children’s emotions, moving from noticing and uncritically accepting them. Through this, the room to satisfy the children’s needs expanded, and these changes in participants led to the increase in psychological stability of their preschool children.

Further, the participants grasped the reality of their situation by staying in the emotion rather than quickly converting their own and others’ negative emotions. Changes in attitudes towards emotions helped improve negative parenting behaviours in response to such emotions and were linked to changes in preschool children. These results are consistent with the assertion that accurate recognition of events that trigger negative emotions helps improve the ability to rationally cope with and process negative situations [35], and that tolerance for negative emotions is formed by no longer avoiding them [36].

The core principle of emotion coaching is to identify the events that cause negative emotions without reducing, repressing, or distorting the emotions, which helps to process these emotions rationally and improve coping skills [13]. This principle of emotion coaching appears to have changed the participants’ perceptions and coping strategies related to negative emotions. The results of this study can be used as empirical basis for establishing the importance of assessing and improving mothers’ emotional intelligence in overdependence on smart devices [12]. This study provides important implications for the importance of emotional intelligence in the field of overdependence where the importance of emotions and feelings is emphasized.

As we used a mixed research method, we could closely check the experience and effects of participating in the programme. Changes in the mothers who participated in emotion coaching group programme can be seen as a psychological process. Our study is significant in that it specifically presented the process and principles of emotional intelligence improvement using qualitative research methods.

### Effects on improvement of mothers’ positive parenting behaviour

We observed a statistically significant change in mothers’ positive parenting behaviour at the time of the post-test, but no significant change continued at the time of the follow-up test. These results are different from the report showing significant changes in positive parenting behaviour by providing emotion coaching group programme to mothers [37]. In this study, both the experimental and control groups participated in the screening test as part of their recruitment and were informed about the level of their children’s smart device use in a supportive way. In addition, the control group was informed that they could participate in the programme if they wished after the study was finished. Based on this, it was estimated that having supportive guidance on the child’s overdependence level from an expert and receiving psychological support had a positive effect on parenting behaviours, as opposed to mothers being vaguely anxious about their children’s use of a smart device. However, it is still difficult to find a previous study that has specifically identified the effect of the mother’s awareness of overdependence on her parenting behaviour; thus, additional research is needed.

Through qualitative analysis, we confirmed that the participants in the experimental group applied compassionate parenting to their children. In other words, unlike before the intervention, they practiced communication in which they regulated and expressed their emotions appropriately. In addition, even in busy daily life, through play, children’s interest in smart devices was naturally diverted. In other words, the participants understood the value of play time with their children and changed their priority in time management.

Unlike fun through smart devices, the play time provided by the mother with increased psychological stability had more meaning than simply diverting attention and providing pleasure. In other words, playing with the mother contributed to the enhancement of the psychological stability of preschool children by relieving negative emotions such as tension. These results are supported by the argument that preschool children form beliefs about relationships through play, and that beliefs develop into trust and stability in themselves and society [13]. This is also consistent with the argument that it is more desirable to switch to other activities in order to persuade preschoolers out of their overdependence on smart devices than to show anger or threaten them [12]. This suggests that additional time and effort are needed to resolve negative emotions in preschool children. Establishing regular play time with children will not only improve attachment by enhancing the psychological stability of preschool children but also contribute to reducing overdependence on smart devices.

Participants with increased recognition and confidence in their emotions practiced authoritative parenting, which clearly set limits for unacceptable behaviours rather than unconditionally allowing their children’s demands. This led to the supervision of children’s use of smart devices. As such, the mother’s authoritative parenting behaviour increases the child’s desirable behaviour through supervision of the child [12]. However, unlike before, in supervising the time and content of smart device use, the level of use was determined through coordination rather than unilateral coercion. In addition, there was a change in waiting for children to give them opportunities to finish using smart devices themselves. These results reflect that the psychological stability of the participants increased based on the improvement of emotional intelligence. The sensitivity to the emotions and desires of the children also improved. Our results suggest ways to help preschool children improve their autonomy and ability to regulate smart devices on their own. A parenting style that acknowledges the appropriateness of emotions would promote the formation of sincere relationships between parents and children [38]. Desirable handling of negative emotions is an important factor in alleviating psychological problems [25].

### Effects on improvement of mothers’ negative parenting behaviour

The results also revealed a significant change in mothers’ negative parenting behaviour at the time of the post-test, but not at the time of the follow-up-test. Direct comparison is difficult owing to differences in subjects, but our finding is different from the report showing changes in mothers’ dysfunctional parenting behaviour after a six-hour emotion coaching group programme to a group of mothers of children with attention deficit/hyperactivity disorder and oppositional defiant disorder [39]. The reasons include the following. First, one participant reported experiencing continuous anger in the situation where the child requested to play, owing to the burden of parenting. Meanwhile, the COVID-19 pandemic has increased the time spent at home [40]. In the age of nuclear families, mothers who lack coping resources tend to show negative parenting behaviours, such as suppressing or neglecting their children in response to parenting stress [41]. Parenting stress experienced by mothers is increased by excessive physical work [41] and exacerbated when emotional support cannot be expected from a spouse [42]. As such, the excessive physical and emotional burden of parenting by the mother increases negative parenting behaviour.

Second, participants’ awareness of negative parenting behaviours may increase after participating in this program. From our qualitative analysis, the theme ‘feelings touched by reflection’ revealed that participants felt guilty when they expressed anger towards their children, which was aggravated when they failed to realize their ideal parenting. Therefore, the participants could have reported a low self-evaluation of their parenting behaviour owing to the increased arousal of negative parenting behaviour. This discussion is based on the report that mild guilt enables tolerant parenting, but excessive guilt causes neglect or inconsistent parenting behaviour and increases when ideal parenting behaviour is attempted [43]. Based on this, interventions aimed at improving mothers’ parenting behaviours should present specific and realistic expectations for desirable parenting behaviours, considering that negative emotions, such as guilt, may occur. In other words, interventions should be structured to help participants set goals for change that consider their physical limitations and experience a sense of accomplishment.

In this study, we provided the intervention for mothers, because most of the main caregivers in Korea are mothers, owing to the cultural characteristics of Korea. However, to reduce the burden of childrearing on mothers, fathers should share in childrearing and provide emotional support [41, 42]. Today, when the low birth rate has emerged as a social problem, social support is also required to alleviate the burden of childcare on parents [41]. Meanwhile, as confirmed through the qualitative analysis, as the psychological stability and responsibility of preschool children increased, the mothers felt that childrearing became “easy,” suggesting that the improvement of emotion coaching-type communication skills is one way to reduce the burden of childrearing on mothers.

### Effects on improvement of mothers’ attachment

Our study found no significant difference over time in the attachment stability of preschool children to their mothers in the experimental group compared with the control group. However, there was a significant positive change in attachment instability at the time of the post-test in the experimental group, and this continued even at the time of the follow-up test. Thus, the emotion coaching group programme has a continuous and positive effect on the improvement of attachment instability. Our results are in line with previous research that showed significant effects on child attachment enhancement by applying emotion-focused parenting interventions to parents [44].

Given that positive parenting behaviours were improved in the control group at follow-up, attachment instability is difficult to ameliorate by strengthening positive parenting behaviours alone, but can be improved by improving negative parenting behaviours. The findings of this study are supported by the argument that attachment between parents and children does not simply emerge from spending time together everyday, but needs to be continuously and actively developed in a systematic and intensive way [38].

According to our qualitative analysis, children with a sense of psychological stability and responsible behaviour can emerge when the attachment of preschool children is improved. Specifically, through the increased sense of security and enriched emotional expression of the child, the mother’s participation in the programme could enhance not only the psychological stability of preschool children but also the expression of affection, such as physical contact, emotional expression, and language expression. Thus, preschool children with attachment damage show psychological instability and disorganized characteristics, whereas children with secure attachment use their parents as a safe base to secure more coping mechanisms and more complex coping and communication skills [45].

Our study used a variety of teaching methods, including lectures as well as participatory activities such as group discussions, role plays, and supervision. This was consistent with reports that passive learning methods, such as lectures, contribute to participants’ knowledge acquisition, whereas active learning methods, such as group discussions, role-playing, and supervision, enhance their applied skills [46]. As such, this study increased the effectiveness of the intervention by using various techniques according to the purpose of the programme.

The participants also contributed to such effectiveness by empathizing with the child by putting themselves in the child’s shoes. In addition, because we allocated four to five participants per group, we could secure the individual training time for the participants. The composition of emotion coaching group programmes should consider the number of participants. Nonetheless, some of our participants were concerned about the stigma on their children owing to their participation in the programme. Thus, interventions for mothers of preschool children who are overly dependent on smart devices must guarantee anonymity and select an independent location to prevent stigma.

The development of emotional intelligence affects quality of life [47]. In addition, since the development of emotional intelligence in preschool children is easily influenced by mothers [27, 48], the improvement in mothers’ emotional intelligence, as confirmed in our study, has long-term effects on not only mothers but also future children. It will contribute to the healthy growth and quality of life of preschool children. The improvement in consciousness of preschool children—who are overly dependent on their mother and smart devices—to feel their emotions freely is seen as an improvement in their ability to regulate their future in a rapidly changing and uncertain world [38].

### Implications

From the synthesis of our results, we generated the following nursing application points. To reduce overdependence on smart devices in preschool children, mothers can seek an assessment of their emotional intelligence, a variable that reflects their psychological stability and emotional sensitivity, and participate in interventions to improve it. In addition, mothers’ ability to apply emotion coaching-type communication should be improved, for them to be able to convey their feelings and thoughts in a desirable way, identify and satisfy their children’s needs, and present clear limits on unacceptable behaviours.

Regular play with a mother with improved psychological stability also contributes to the enhancement of attachment and reduction of overdependence on smart devices in preschool children. Thus, interventions to assess and improve the play time and content between mother and preschool children are required. To increase mothers’ psychological stability and reduce the burden of childrearing, spouses should offer appropriate support. Moreover, social support for parents raising preschool children is needed.

### Limitations

This study has several limitations. As the intervention providers and data collectors in this study are the same, the possibility of a Hawthorne effect cannot be ruled out. Future research may conduct a randomized assignment design to generalize the study results. In our study, changes were measured using mothers’ self-reported assessment. To objectively evaluate the intervention effect, researchers should use observation evaluation by a third-party researcher or physiological indicators, such as cortisol or oxytocin, which can be used to evaluate stress or emotional changes as indicators reflecting psychological stability [49].

## Conclusions

Mothers who are concerned about their preschoolers’ excessive use of smart devices are more likely to experience negative emotions, such as anxiety and anger, in the process of regulating the usage. The emotion coaching group programme delivered in this study was effective in helping mothers understand the importance of emotions and focus on emotions rather than their children’s overdependence. It helped mothers to improve their emotional intelligence by making a conscious effort to be sensitive and empathetic to their own and their children’s emotions. In addition, improving their emotional intelligence helped mothers to practice an authoritative yet gentle positive parenting behaviour. The increased play time with the changed mother helped improve attachment stability in the child by increasing their psychological stability. This led to positive effects in reducing the preschoolers’ overdependence on smart devices. Active use of the results of our study will contribute to the growth and development of preschool children and the improvement of their quality of life.

## Data Availability

All data generated or analysed during this study are included in this published article.

## List of abbreviations

AEQT: Adult Emotional Quotient Test
GEE: Generalized Estimation Equation
MCAS: Mother–Child Attachment Stability
MPBQ: Maternal Parenting Behaviours Questionnaire
SO: Smart device Overdependence
SOS: Smartphone Overdependence Scale
